# Effects of Wastes from the Brewing Industry in Lightweight Aggregates Manufactured with Clay for Green Roofs

**DOI:** 10.3390/ma10050527

**Published:** 2017-05-15

**Authors:** Romina D. Farías, Carmen Martínez García, Teresa Cotes Palomino, Myriam Martínez Arellano

**Affiliations:** Department of Chemical, Environmental and Material Engineering, Higher Polytechnic School of Linares, University of Jaen, Scientific and Technological Campus of Linares, 23700 Linares (Jaén), Spain; romina.d.farias@gmail.com (R.D.F.); mtcotes@ujaen.es (T.C.P.); mma00037@red.ujaen.es (M.M.A.)

**Keywords:** brewing industry waste, porosity, insulating properties, green roofs

## Abstract

This study investigates the effects of sieved wastes generated from the brewing industry on lightweight aggregates manufactured with clay. Sludge from a wastewater treatment plant, bagasse and diatomaceous earth were used to obtain the samples. These wastes are usually dumped in landfills, but the current increase in restrictions on dumping and interest in improving the environment make our proposal for gaining value from these wastes a significant contribution. Laboratory tests show that the new aggregate has low bulk density and increased water absorption and porosity. The thermographic camera results provide evidence that new aggregates have significant insulating properties and are suitable for use on green roofs.

## 1. Introduction

Aggregates, granular materials added as dispersed phase for mortar and concrete, usually consist of crushed rocks. They tend to be porous materials of natural origin or materials artificially manufactured to have low density. Lightweight aggregates’ high porosity gives them their properties of low density, frost resistance, and high thermal and acoustic insulating power [1].

These materials are manufactured industrially through a process of thermal transformation from natural raw materials (clay, volcanic glass, shale, slate, and vermiculite) or industrial wastes. In this field, the production of lightweight aggregates has been studied with different wastes such as fly ash, bottom ash, sewage sludge ash, combustion ash from solid waste, blast furnace slag, recycled glass, mining waste, sludge and contaminated soils [2,3,4,5,6].

Lightweight aggregates can be manufactured by two processes: cold bonding pelletization and sintering at high temperatures. The former, which has been the subject of intensive research in recent years, consists of a process of cement-based solidification/stabilization that enables valuation of the waste to obtain a material that is safe for manufacturing construction materials [7,8,9].

This manufacturing method also has economic and environmental advantages, as it occurs at room temperature and does not produce secondary pollution.

Natural raw materials or industrial by-products are used in the thermal transformation process. The process is carried out in rotary kilns at maximum temperatures of 1000–1300 °C.

Thermal expansion is the method most widely used to fabricate highly porous lightweight aggregate on an industrial scale. The raw materials are first crushed to a particle size suitable for thermal processing. Once dry, the material is subjected to heat treatment for a short time, during which the particle softens and undergoes expansion caused by internal gas evolution. Upon cooling, the particles retain hardened porosity caused by gas bubbles [10]. 

The products are used as insulation in industrial plants [11,12,13] or as lightweight aggregate in mortars and concretes [14,15,16]. Other authors have studied the thermal performance of green roofs against three existing roofs [17]. The present study proposes using the lightweight aggregates manufactured with clay and waste from the brewing industry as insulation in building roofs.

The high demand for building materials has significantly diminished natural stone reserves, causing irreparable damage to our environment, and the concrete industry is largest consumer of limited natural resources such as water, sand, gravel and crushed rock [18]. 

The construction industry alone consumes more raw materials than any other economic activity, hence the need to develop sustainable materials. The best alternative for sustainable development of the concrete industry is use of waste materials and wastes rather than raw materials [19].

Prior research shows that using lightweight aggregates (expanded perlite) in concrete mixtures can lower thermal conductivity about 0.13 W/mK [20]. By using lightweight aggregates prepared with expanded polystyrene waste to replace the natural aggregates, the concrete´s thermal conductivity was reduced to 0.600 W/mK [21]. This decrease in thermal conductivity suggests to the potential of applying these materials to insulate buildings or form part of green roofs.

The main factors causing today’s energy problems are the higher energy demands of developed societies and the growing demand by developing countries, as well as limited energy sources and the environmental burden generated by their use. As the thermal demand of residential and commercial buildings accounts for about 40% of total world energy consumption [22], any improvement in energy efficiency (conservation and energy saving), represents a benefit to the global environment and to the economies of developed and developing countries. Given this situation, Spain, and to a greater extent northern European countries, has developed a trend towards bioclimatic architecture, which adapts energy to geographic location and uses of renewable energy.

Green roofs are a system of multilayer roofs that permit propagation of vegetation on an exposed surface while ensuring the integrity of the lower layers and the roof structure. These roofs provide a set of additional features and are designed and built with relatively simple technical parameters, not requiring expensive or highly specialized technologies.

This practice could help to reduce building energy consumption and improve the thermal environment of buildings, reducing the temperature inside the building [23,24]. Numerous studies have thus been performed to determine thermal behavior based on the type of building cover and layer thickness of the substrate on which the vegetation grows [17].

**Figure 1:**
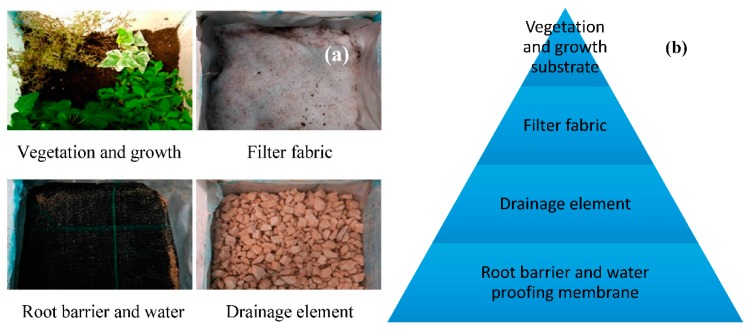
(**a**) Layers used in a Conventional Green Roofs; (**b**) Green Roof Scheme.

Figure 1a shows the multilayer of green roofs. This study proposes to employ the lightweight aggregates manufactured with clay and waste from de brewing industry in the drainage layer instead of using traditional product as expanded clay or crushed brick. Figure 1b shows a scheme with distribution of green roof layers.

Roofs have different cropping systems. The intensive system, or conventional gardens, is accessible and has thick substrates that host a variety of plants. The substrate layer must be deeper than 30 cm, which increases the system weight. This type of system is better suited to new constructions, as it is necessary to perform a detailed structural analysis since the system weighs over 250 kg/m^2^.

Intensive systems are low maintenance and are usually installed in inaccessible places. Often, species are planted that require little moisture, have only 5–15 cm substrate, and subsist on rainwater. As the approximate system weight is 60 to 140 kg/m^2^ [25], this type of green roof is most suitable for use in existing buildings, as it requires minimal structural reinforcements to support the additional weight. These lightweight aggregates are appropriate because they decrease the load that the building structure must bear.

This study uses waste from the brewing industry. In the food industry, the brewing sector has a strategic economic position, with an annual production in Europe of 390 million hectoliters in 2011. Beer is the fifth-most-consumed beverage in the world after tea, carbonated beverages, milk and coffee [26].

At present, there is great political and social pressure to reduce pollution from industrial activities. Almost all countries, developed and developing, are trying to adapt to this reality by modifying their processes to recycle waste. Consequently, most large companies no longer consider waste as a waste, but as feedstock for other processes. Compared to the industries in this study, the brewing industry generates relatively large amounts of waste. Bagasse, hops and yeast wastes, and diatomaceous earth are the most common. These wastes are currently reused as agricultural products [27], and bagasse is the most abundant brewing waste, generating approximately 20 kg per hectoliter of beer produced [28].

In the brewing industry, organic products constitute the largest group of waste by volume generated. Some studies have characterized beer industry wastes, investigating the effects of adding spent malt grains in brickmaking [29]. Although most of the specialized literature on obtaining light gravel through the process of sintering has focused on using inorganic natural waste, some studies treat the use of organic waste, primarily from agriculture and forests [30,31]. This organic waste has significant calorific power comparable in some cases to that of solid fuel, an additional advantage in sintering the light gravel, since part of the energy needed for the thermal process is provided by the waste. This means energy savings and a reduction of polluting emissions relative to the process of manufacturing gravel from natural raw materials or inorganic wastes. Several studies analyze the impact of different pore-forming agents on the physical, mechanical and thermal properties of clay bricks [32].

In the case studied here, the beer industry, sludge from a wastewater treatment plant is used to produce this type of material. The results show a linear relationship between the amount of waste added and water absorption values [33,34].

This paper focuses on development of lightweight aggregates using brewery waste (bagasse, diatomaceous earth and wastewater treatment plant sludge) combined with clay. The materials were shaped by pelleting to obtain materials with low density and good insulating properties.

## 2. Materials and Methods

### 2.1. Raw Materials

**Figure 2:**
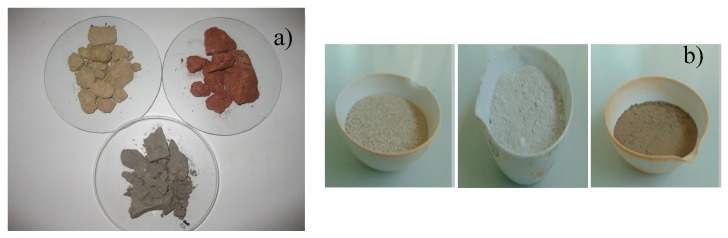
Raw materials: (**a**) Clay (Yellow, red and black); and (**b**) Wastes (Bagasse, diatomaceous and sludge).

The raw materials used in this study are three types of clay—red, black and yellow—from the ceramics industry in the city of Bailén, in the province of Jaén (Spain), as well as bagasse, diatomaceous earth, sludge from a wastewater treatment plant from the brewing industry, and water from the general utility network. Figure 2 shows raw materials.

Because the clay received in the laboratory had a very heterogeneous grain size, it was subjected to milling in a hammer mill. If the clay is shaped by pressure, it must subsequently be passed through a 0.5 mm sieve. Control of particle size is important, as it influences the physical and mechanical characteristics of the final product.

The brewing industry waste was collected at the plant and transported to the laboratory. It was then dried in an oven at 90 °C for 24 h to reduce the high initial humidity and subjected to grinding in the hammer mill. The resulting substance was transferred to an agate ball mill and sieved to obtain proper grain size.

### 2.2. Characterization of Raw Material

The raw materials were tested to determine their physical and chemical characteristics, as follows:

- X-ray diffractometry of clay and wastes was performed using an automatic Siemens D5000 diffractometer (Siemens AG, Munich, Germany), with Bragg-Brentano geometry (θ/2θ) and Kα1, 2 radiation, equipped with a graphite monochromator to eliminate Cu Kβ radiation.
- Chemical composition was determined by X-ray Fluorescence (XRF) using Philips Magix Pro (PW-2440) equipment (Philips, Amsterdam, The Netherlands).
- The total carbon, hydrogen, nitrogen and sulfur contents were determined by combustion of the samples in an O_2_ atmosphere using the CHNS-O Thermo Finnigan Flash Elementary Analyzer EA 1112 (Thermo Scientific, Waltham, MA, USA). The determination of the organic content was performed according to ASTM D-2974 [35].
- The Higher Heating Value (HHV) and Lower Heating Value (LHV) were determined by a Parr 1341 Plain Oxygen Bomb Calorimeter (Parr Instrument Company, Moline, IL, USA), following the UNE 32 006:1995 standard [36].
- Thermal behavior was determined by Thermogravimetric Analysis (TGA) and Differential Thermal Analysis (DTA) using a Mettler Toledo 850e balance (Mettler Toledo, Columbus, OH, USA). Samples of approximately 15–30 mg were placed in a platinum crucible and heated at a rate of 10 °C/min in the range from room temperature to 900 °C. Air flow the samples was maintained at a rate of 50 mL/min. The data shown reflect weight loss that depended on temperature and DTA diagram simultaneously.

### 2.3. Preparation of Samples: Sintered Materials

The dried wastes were mixed with the three types of raw clay in equal parts: red, black and yellow (Mixing Clays, MC). The appropriate quantities of dry waste and clay were weighed and then mixed thoroughly in a mortar. The samples were shaped by pelletizing. To determine the effect of organic waste material on the clay-matrix, different amounts of waste (0–15% wt) were added to the clay. Waste-free test samples (clay only) were made as a reference. Water was added during this process to obtain the right plasticity, and the samples were shaped, measured and weighed. 

The samples were then fired at 110 °C for 48 h to reduce moisture content. As the drying progressed, the particles came closer together and contraction increased [37]. 

**Figure 3:**
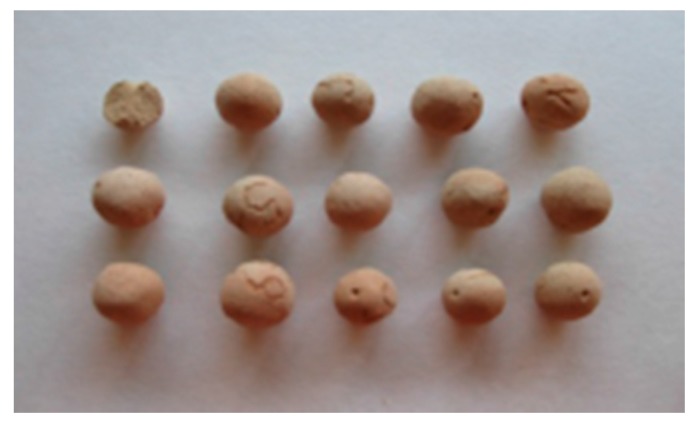
Sintered Materials.

To produce the final resistance and cohesion of the material, the dried samples were subjected to a firing process in a kiln that sintered the grains and changed their density and porosity. Before being loaded in the oven, the samples were preheated into the oven at temperatures near 200 °C for 24 h to prevent fractures during the sintering process. Once the kiln reached the desired temperature (900, 950 or 1000 °C), the samples were introduced. They remained in the oven at the required temperature for 1 h, after which the kiln was turned off and they were allowed to cool through natural convection (Figure 3). 

### 2.4. Characterization of Sintered Materials

The physical properties of the sintered materials were determined as follows:

- Weight loss from sintering was obtained by determining the difference between weight after the drying stage at 110 °C and after the firing stage at 900, 950 and 1000 °C.
- Adsorption and desorption isotherms with N_2_ were determined and distribution of mesoporosity studied following the BJH (Barrett, Joyner, and Halenda) method, using an ASAP 2020 Accelerated Surface Area and Porosimetry System by Micromeritics (Micromeritics, Norcross, GA, USA). The following parameters were measured with the adsorption isotherm, using the BET surface area method (m^2^/g), pore volume (cm^3^/g) and pore size (Å).
- Water absorption capacity is defined as the measurement of moisture when a solid is completely immersed in water for a long time. The test to determine water absorption capacity was implemented according to Standard Procedure ASTM C373 [38].
- Absolute density was determined using a Gas (He) Displacement Pycnometry System AccupycTM II 1340 by Micromeritics (Micromeritics, Norcross, GA, USA).
- Insulating properties were determined using the thermal imaging camera Fluke Ti-32 (Fluke, Everett, WA, USA).
- The microstructure of the formed samples was observed by means of a scanning electron microscope (SEM), with a high-resolution transmission electron microscope JEOL SM 840 (JEOL, Solutions for Innovation, Peabody, MA, USA).

Life Cycle Assessment (LCA): LCA has been analyzed, comparing the environmental impact generated in terms of carbon footprint, from lightweight aggregates elaborated by the use of different percentages of wastes and produced without them. Being interested in the contribution in terms of greenhouse gas given by the production of aggregates, the chosen calculation method was the “IPCC 2013 GWP 20y”, developed by the expert judgment from the Intergovernmental Panel on Climate Change (IPCC). It contains in particular the climate change factors defined by the IPCC considering a timeframe of 20 or 100 years. The Ecoinvent and Data Archive database, provided by *Sima Pro 8 software* (Version 8, Pré Sustainability, Amersfoort, The Netherlands), developed by Pré Consultants, has been used.

## 3. Results and Discussion

### 3.1. Raw Materials

#### 3.1.1. Chemical Composition and Loss on Ignition

Table 1 presents the chemical composition and loss on ignition (L.O.I.) of the clay and the waste ash.

The largest component of the clay mixture employed is found to be silica (54.26%), followed by alumina (Al_2_O_3_), the content of which is within the range of values for clays used in masonry. All other components—such as Fe_2_O_3_, MgO, CaO, Na_2_O, K_2_O, and TiO_2_—are within the levels established for illite clays used in the ceramics industry. 

Bagasse ash is rich in silica (44.33%) and phosphorus (21.29%) and contains large amounts of calcium, magnesium and potassium. Sludge ash has high percentages of silica, with a content of 44.33% and calcium oxide (26.45%), with contributions from other elements, such as aluminum and phosphorus oxides. The diatomaceous waste ash is composed mainly of silicon oxide, with a content of 67.34%, and much lower amounts of aluminum oxide (4.95%), sodium and potassium, the latter two considered as fluxing agents.

#### 3.1.2. Total Content of Carbon, Hydrogen, Nitrogen and Sulfur: Heating Values

The total content of carbon, hydrogen, nitrogen and sulfur in the clay and wastes are shown in Table 2.

The mix clays presented a LOI of 11.90 mass% and a lower content of organic matter determined according to the ASTM D-2974, 3.84%. However, the high carbon and hydrogen bagasse content provides high calorific value, 4762.47 kcal/kg, comparable to that of alternative fuels, for example biomass or urban wastewater sludge, with HHV between 3576 and 3800 kcal/kg [39]. Neither raw material shows presence of sulfur, an important result given that sulfur is an undesirable compound because this compound could generate pollutants in the combustion process.

#### 3.1.3. Thermogravimetric Analysis (TGA) and Differential Thermal Analysis (DTA)

**Figure 4:**
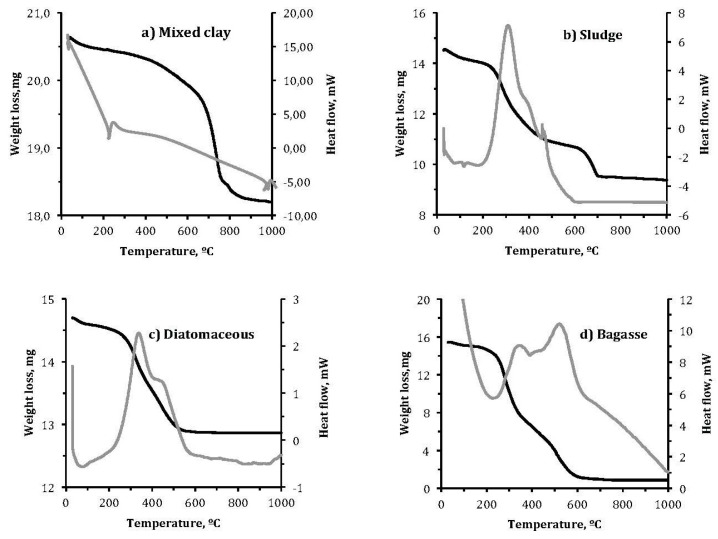
Thermogravimetric Analysis (TGA) and Differential Thermal Analysis (DTA) curves of: (**a**) mixed clay; (**b**) sludge; (**c**) diatomaceous; and (**d**) bagasse.

The thermal behavior of clay and wastes was analyzed by TGA and DTA, as shown in Figure 4. The clay mixture produces a small endothermic peak at 51.4 °C that can be attributed to moisture loss, resulting in 2% mass loss (Figure 4a). As temperature increases, weight loss increases. 

At 200 °C, another major endothermic peak is observed, as above. It corresponds to the elimination of interlaminar absorbed water present in the phyllosilicate clays. As the temperature increases above 200 °C, the mixture begins to oxidize organic matter, which appears as an exothermic peak. In addition, dehydroxylation of the clay mineral occurs from 500 °C, also causing mass loss. Between 700 and 850 °C, decomposition of carbonate clays occurs, producing calcium oxide and carbon dioxide, as evidenced by the endothermic peaks of ATD. Between 900 and 1000 °C, peaks associated with the formation of calcium silicates such as anorthite and helenite appear (Figure 4a).

In the sludge, several small endothermic peaks are observed before 200 °C, where the weight loss is not very pronounced, due to elimination of water. From 350 to 700 °C, the marked weight loss corresponds to the combustion of organic matter. After 700 °C, weight loss stabilizes (Figure 4b). In the case of the diatomaceous earth, total weight loss is less pronounced, amounting to 1.5%. At 150 °C, another endothermic peak can be observed, which corresponds to the removal of moisture from the residue. The loss of the small amount of organic matter represents a small energy input that results in an exothermic peak at 350 and 500 °C (Figure 4c).

The bagasse shows an endothermic peak due to loss of non-interstitial binary hydrides and two large exothermic peaks between 300 and 600 °C related to the significant weight loss that occurs in this interval due to combustion of the organic matter in the bagasse (Figure 4d). The peaks indicate the energy released during combustion of the organic matter. These results agree with those obtained for the calorific value.

### 3.2. Sintered Materials

#### 3.2.1. Weight Loss

Table 3 shows the results of weight loss determination. Weight loss on ignition measures the difference in sample weight before and after the firing process. According to the results obtained for elemental analysis and the calorific value, weight loss increases, as does waste content. Further losses are observed in the residue with higher organic content. Bagasse shows a residue loss of 9.8–22%; followed by sludge, with losses of 9.8–14%; and finally diatomaceous earth, with lower losses of 9.8–10.7%. Comparing the data as a function of temperature shows that the differences do not exceed 1% for the same percentage of waste added. Thus, Temperature is not a determining factor in the test. These results agree with those obtained for the DTA/TGA, where no appreciable weight loss occurred between 900 and 1000 °C in any of the waste.

#### 3.2.2. Adsorption and Desorption Isotherms

**Figure 5:**
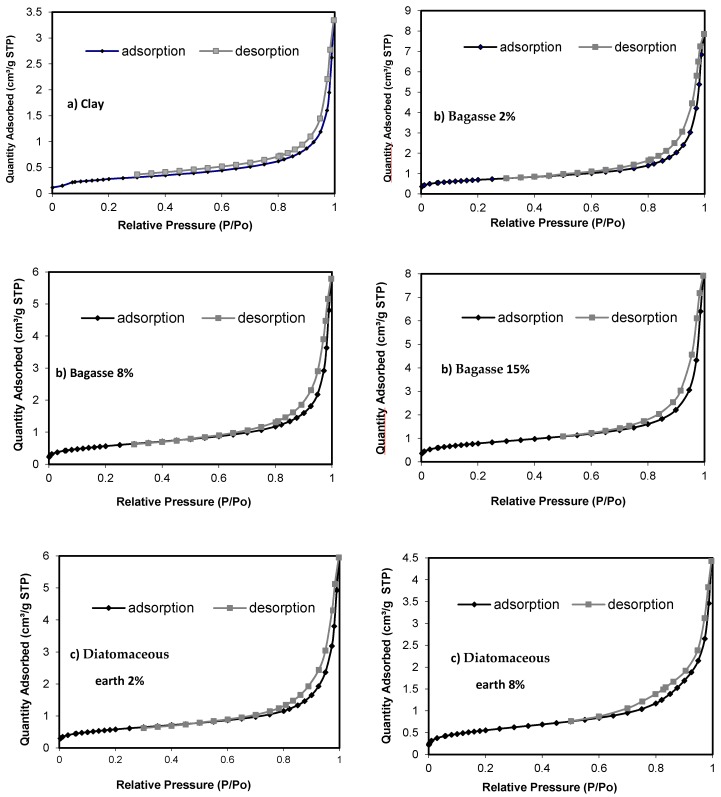

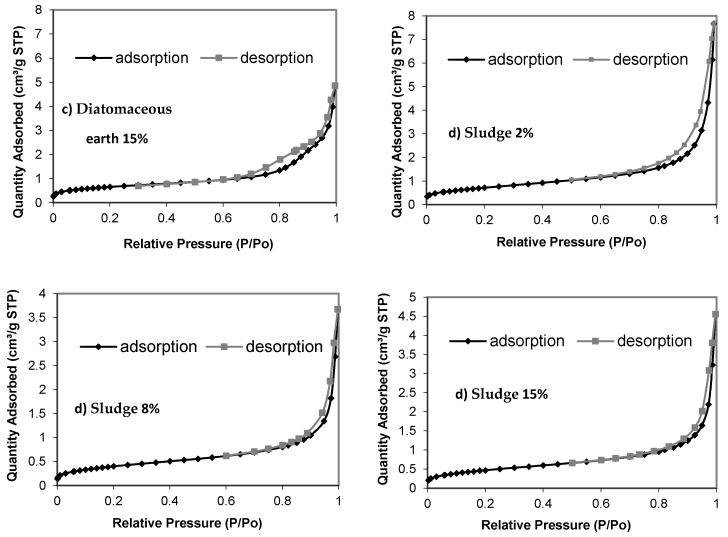
Adsorption Isotherms, N_2_ 77 K: (**a**) Clay; (**b**) Bagasse; (**c**) Diatomaceous earth; and (**d**) Sludge (950 °C).

Figure 5 shows the results of the adsorption isotherms. All isotherms obtained are Type IV, with their distinctive hysteresis loop characteristic of mesoporous solids, materials with a pore size of 20–500 Angstroms. The hysteresis loop indicates that the mesopores are filled by capillary condensation and the percolate properties of the solid. The figure shows a type H3 hysteresis loop following the IUPAC classification. Such bonds are obtained when working with clusters of parallel plate pores (slit-shaped). Significant mesoporosity is present, as a large quantity of nitrogen is adsorbed at medium and high relative pressures [40]. The presence of hysteresis and an increase in the amount of nitrogen adsorbed at these relative pressures also indicate high mesoporosity (Figure 5a–d) [41]. Distribution of mesoporosity was studied using the BJH (Barrett-Joyner-Halenda) model for the adsorption branch with the Halsey equation and Fass correction. 

The clay samples of bagasse and diatomaceous earth showed (Figure 5a–c) no significant absorption at relatively low pressures, indicating the presence of micropore distribution studied by Brunauer’s model MP [42,43].

**Figure 6:**
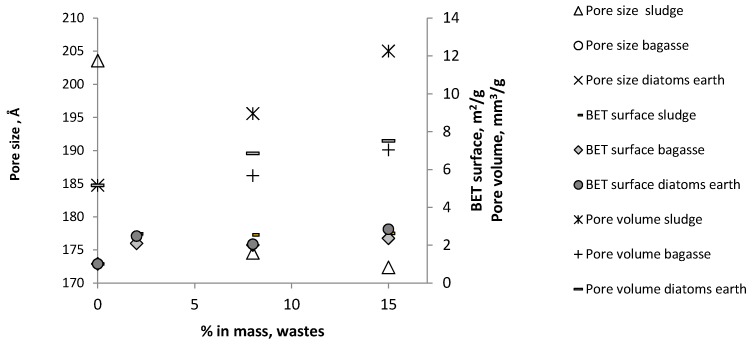
Size distribution and pore volume. Brunauer-Emmett-Teller surface, BET surface, 950 °C.

Porosity, pore size, volume and evolution of Brunauer–Emmett–Teller surface (BET surface, m^2^/g) are shown in Figure 6. As a higher percentage of waste is added, more BET surface and pore volume were generated. The incorporation of 15 mass% waste was produced the higher values for BET surface: 2.8455, 2.6266 and 2.3681 m^2^/g for diatomaceous, sludge and bagasse, respectively. Pore size decreasing relative to the samples without residue, the incorporation of 0 mass% sludge produced the higher value, 203.58 Å, decreasing to 174.56 Å when 8 mass% is incorporated and 172.37 Å when 15 mass% sludge is incorporated (Figure 6). Higher pore surface values were also obtained for the samples with the highest organic residue content: bagasse.

#### 3.2.3. Technological Properties

Table 4 shows the results of the properties determined by the ASTM C373: water absorption, bulk density and open porosity. In the case of the expanded aggregates prepared for use in green roofs, high absorption values were linked to higher percentages of open porosity and lower bulk densities, unlike the properties of other ceramic construction products. Bulk density and open porosity are directly related to waste content added but these properties showed inversed trend in the overall compositions and temperatures studied, obtained for both the best results for sintered process at 1000 °C. The incorporation of the wastes produced a decreased in the bulk density of the clay (1698.8 kg/m^3^). The organic matter in the wastes was transformed during the thermal process and it leads to an increase in the open porosity of the ceramic bodies. The incorporation of 4, 6, 8, 12.5 and 15 mass% wastes produced a decrease in the bulk density obtaining values of 1409.5, 1109.8 and 1255.0 kg/m^3^ bulk density values for samples where 15 mass% sludge, bagasse and diatomaceous have been added, respectively. This is a beneficial property. Lower densities create lighter pieces, preventing overload of the structure. Open porosity shows inversed trend to bulk density, this property increased with the amount of waste incorporated, the values obtained are showed in Table 4. The incorporation of 15 mass% of wastes to the body clay increased to open porosity obtaining a maximum open porosity value of 50% by samples with 15 mass% of wastes and all sintered temperatures. The porosity generated in the combustion of organic matter gives the parts insulating properties, as evidenced in the thermal imaging camera test. This characteristic helps to protect plants and the building from extreme temperatures where employed. On the other hand, water absorption values increased with the amount of waste added and the higher values were obtained for 15% mass of bagasse, medium values of 50% for all sintered temperatures. The samples also show high absorption values conducive to good drainage, thus saving water, as all components can absorb water until saturated, leaving moisture available to the plants and allowing remaining water to drain. Higher water absorption indicates greater open porosity and larger pore size, results consistent with those presented in Figure 6. Low-density values and high values of water absorption indicate porous materials.

In the three wastes used, the same trend of increased porosity and water absorption, as well as decreased bulk density is observed as the percentage of waste added increases. Change in temperature does not affect the results significantly. Table 4 shows that dry bagasse gives the best results for use in green roofs.

#### 3.2.4. Insulating Properties

**Figure 7:**
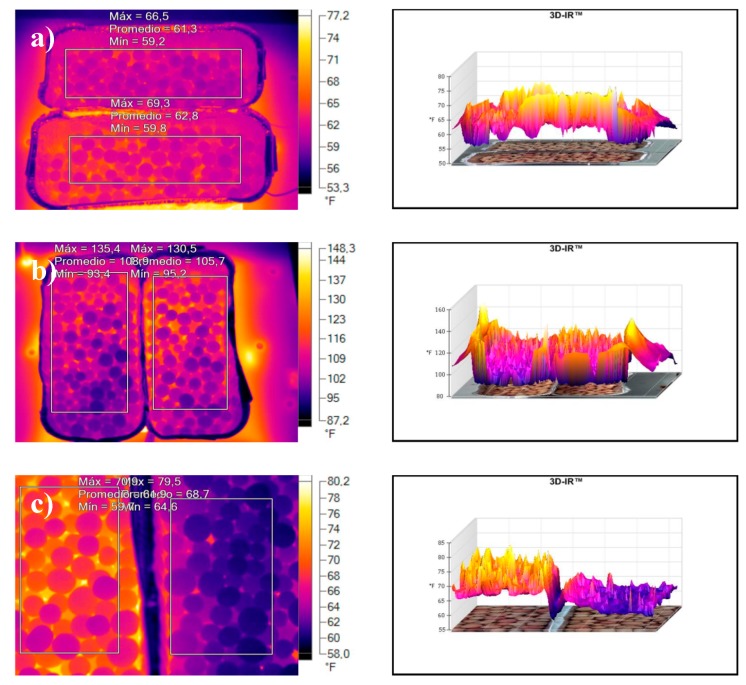
Thermal imaging camera images: (**a**) 15 mass% Diatomaceous earth; (**b**) 15 mass% Sludge; and (**c**) 15 mass% Bagasse.

Figure 7 shows the results of the thermal imaging camera test. The measurements were made with the imager on the surface of a thermal house, placing the aggregate layer on top, the clay only aggregates on the left and the highest percentage of waste on the right, 15% by weight for the three temperatures. According to the results obtained for weight loss and porosity of the bagasse samples, higher organic content can be used effectively to form pores, increase open porosity and decrease bulk density, improving the material’s insulating characteristics. In the case of the bagasse, the temperature drop measured with the thermal imaging camera is 18.24%. This percentage decreases in proportion to porosity values obtained: 4.85% for sludge and 4.10% for diatomaceous earth.

#### 3.2.5. Microstructure

**Figure 8:**
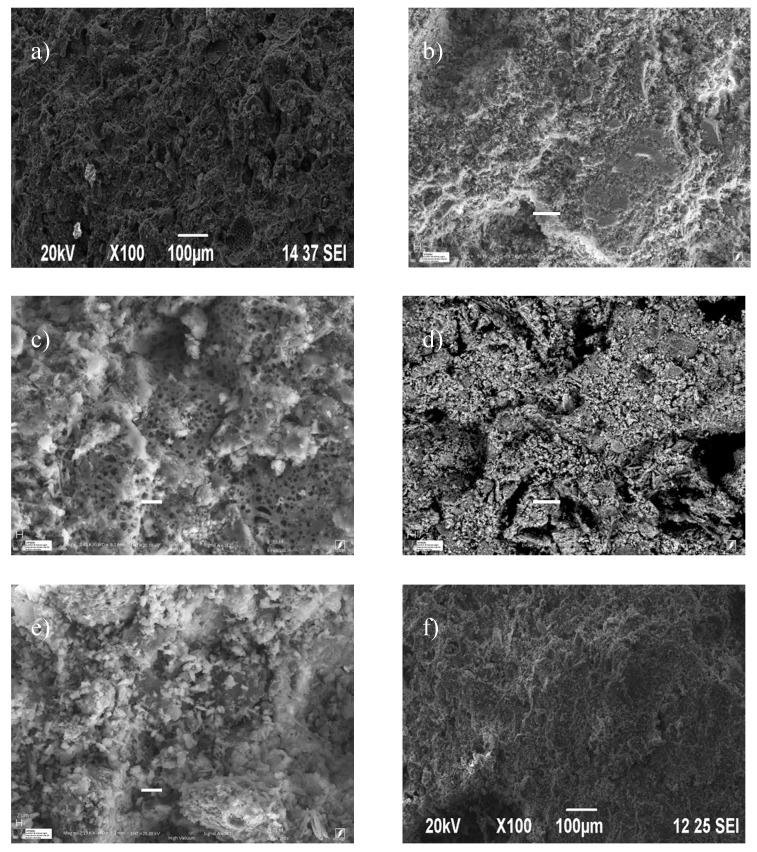
SEM micrographs: (**a**) Clay; (**b**) Bagasse 2%; (**c**) Bagasse 4%; (**d**) Bagasse 15%; (**e**) Sludge 15%; and (**f**) Diatomaceous Earth 15%.

SEM (Figure 8) produced the morphological study of the aggregate containing both clay and mixtures with small and large waste content. As seen in the micrographs, porosity of the samples without waste (Figure 8a) changes when waste is added. Such changes are more noticeable in the bagasse, with its greater organic content. Small amounts of bagasse (2 and 4 mass%) cause increased closed porosity with regular spherical pores (Figure 8b,c), while larger amounts of bagasse (15 mass%) increase open porosity through connection of the macropores, as shown by the data on water absorption and porosity (Figure 8d). The changes are less noticeable in the case of sludge and diatomaceous earth, as shown in the micrographs of samples with clay, 15 mass% sludge (Figure 8e) and 15 mass% diatomaceous earth (Figure 8f).

#### 3.2.6. Life Cycle Assessment (LCA)

In order to carry out the calculation of the carbon footprint, it is necessary to define the concept of Life Cycle Analysis (LCA), which is based on the collection and analysis of the inputs and outputs of a system to obtain results that show the potential environmental impacts to be able to determine strategies to reduce them.

The assessment includes all the activities, processes, and by-products connected to the system analyzed, including raw material processing, production, maintenance, recycling, and disposal (“end of life”). An LCA study is divided into four main areas according to ISO standards UNE-EN-ISO-14040:2006; UNE-EN-ISO-14044, 2006, [44,45]:

- Goal and scope: Definition of system boundaries, functional unit used in the analysis and data sources description.
- LCI: Definition of inputs and outputs, data-based process of quantifying energy, raw material requirements, gas emissions, effluents, solid waste and every environmental release incurred throughout the life cycle processes.
- LCIA: A method, quantitative, and/or qualitative process to characterize and assess the effects of the environmental loadings identified in the inventory component.
- Life Cycle Improvement: A systematic assessment of the needs and opportunities to reduce environmental burdens associated with energy and raw materials use and waste emissions throughout the whole life-cycle of a product process, or activity.

The extraction activities of clay material, drying and sintering of the ceramic samples are the processes with more externalities in terms of environmental impact. 

The impact category analyze was the Global warming Potential (GWP), according to the Method IPCC 2013 GWP 20y, to analyze and compare the environmental impact of the decrease the in energy demand for sintering furnace. The aim of this CFP study is to compare the energy compensation and environmental performance between traditional production, represented with samples firing, and LWAS made with different percentages of organic waste/by-product. 

The calculation of the CFP is part of the LCA, a method by which the environmental externalities generated by a system are based on the contribution of Greenhouse Gases (GHG), emitted by direct or indirect effects. It is a simplified version of the LCA, in which instead of considering several impact categories, only the Global Warming is considered. UNE-EN ISO 14064-1:2012 [46], determines the standards for the calculation of the Carbon Footprint.

It has been compared the environmental impact generated in terms of CFP, from LWAS elaborated by the use of different percentages of technical nutrients and commercially produced, made with 100% virgin raw materials, i.e., clay. The functional unit (F.U.) is 1.00 kg of LWAS thermal treated.

It was decided to include in the LCA the processes of raw material extraction and the sintering process of the samples considered the most relevant in terms of energy consumption and environmental impact generation.

The intermediary processes derived from the preparation of raw materials like crushing, sieving, mixing, water addition, pelletizing and drying processes were considered the same for all in terms of energy inputs and environmental impact, although the nature of the waste are different and may require higher energy consumption in the milling/crushing process. Water addition could also change for mixtures with residues.

The introduction of an organic compound within the mixture allows reducing the input energy to the system. The combustion of the organic substances in fact supports the heat treatment, making it less preponderant the external contribution in terms of heat. On the basis of this assumption, for each process it was necessary to calculated de Higher Heating Value (HHV) of each residue, and considered a reduction in terms of heat input described as its follows: the rotary kiln to produced 1 kg of lightweight aggregates, have an average consumption of 1600 kcal by sintering at 1000 °C. The energy that can be saved in the firing phase (according to HHV showed in Table 2) was for samples with 15% wt of sludge, 186.38 kcal/kg, samples 15% wt of bagasse, 714.37 kcal/kg and for samples 15% wt of diatomaceous, 80.56 kcal/kg.

The development of the LCA model included the establishment of an inventory database containing quantitative and qualitative information about energy use and airborne emissions. Source of data inventory was obtained from Ecoinvent 3, USLCI and EU and DK Input Output Database libraries.

**Figure 9:**
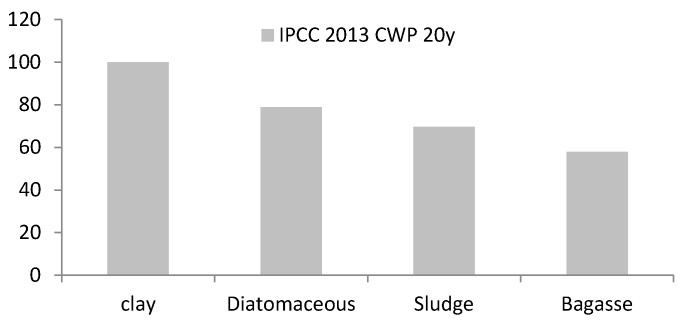
Carbon FootPrint, CFP of lightweight aggregates. Raw material extraction and sintering process in comparison of Clay, 15 mass% Diatomaceous earth, 15 mass% Sludge and, 15 mass% Bagasse. Sintered at 1000 °C.

In Figure 9, it can be seen that the energy intake from residues inserted inside the mixture can bring advantages since combustion reduces the energy necessary to the completion of the heat treatment of the aggregates. The resulting advantage in terms of reduced air pollution is therefore a plus to add to the fact that the production of lightweight aggregates with the use of food waste allows reducing the material to landfill.

Table 5 shows Carbon FootPrint (CFP) in terms of kg CO_2_ equivalent contribution. The use of wastes, thanks to their heat values can contribute to the heat release during the firing stage. CFP calculation provided an understanding approach to determine the specific heat capacities of samples by incorporating sludge, diatomaceous and bagasse. Therefore, samples with 15% by mass of bagasse and sintered at 1000 °C show the lower value, 3.280 kg CO_2_ eq, due to the higher heating values when they are added by self-combustion to the clay mixture so less energy is needed to sintering process. Thus, the addition of these wastes from brewing industry has been demonstrated to be efficient for energy savings in lightweight aggregates manufacturing.

These results accord with other authors that studied the potential environmental effect of waste valorization through the development of ceramic materials compared with traditional ceramic [47,48].

## 4. Conclusions

The results demonstrate the possibility of using waste from the brewing industry, sludge, bagasse and diatomaceous earth in the production of expanded clay-based aggregates, and of using these aggregates for green roofs. The chemical composition, studied by X-ray diffraction, is appropriate for use in preparation of clay-based products. The organic content of the three wastes used benefits the pieces produced as they have lower bulk densities and increased porosity. These pieces could be used to build green roofs. They would not overload the structure and would, as confirmed by the thermal test, improve insulating properties and protect the building and the species that could use the green roof based on its temperature fluctuations. In all three cases, there is a direct relationship between amount of waste added and water absorption and porosity, and an inverse relationship between amount of waste and apparent density.

The results obtained from measuring porosity and thermal imaging reinforce the microstructure observed using SEM: addition of waste increases porosity, a phenomenon more evident in the bagasse.

Lower percentages of bagasse increase closed porosity; as the micrographs show rounded and closed pores, high percentages (15%) show connected pores resulting in a more open structure with interconnected pores.

Absorption properties, bulk density and open porosity are not affected by temperature. The best results were obtained using bagasse in percentages of 10–15%. At higher values, high values for absorption and porosity result in defective parts.

In addition, LCA results show that wastes from brewing industry in combustion process releases CO_2_ emissions that can be recaptured and regrown by photosynthesis. Results have confirmed adding these wastes into clay body lead to decreasing the firing rejects. The recycling of these wastes as a lightweight aggregates seems to be feasible solution, not only for the pollution problem but also an economical option to produce green roofs.

It is shown that the calorific value of wastes, to a greater extent in the case of bagasse by burning, generates an exothermic reaction, which lowers power consumption in the sintering process. This contribution produces energy savings in the furnace energy requirements, resulting in lower gas emissions to the environment, therefore economic savings. However, it is important to note that the LCA results are very preliminary and will need to delve into this aspect.

## Figures and Tables

**Table 1:** Chemical composition of the clay and waste ash.

Component	SiO_2_ (%)	Al_2_O_3_ (%)	Fe_2_O_3_ (%)	MnO (%)	MgO (%)	CaO (%)	Na_2_O (%)	K_2_O (%)	TiO_2_ (%)	P_2_O_5_ (%)	Zr (ppm)	LOI
Red Clay	50.15	19.67	8.21	0.12	3.57	2.80	0.02	6.37	0.83	0.16	135.5	7.86
Yellow Clay	60.40	11.46	4.02	0.03	1.39	8.73	0.38	2.73	0.64	0.10	270.6	9.54
Black Clay	55.98	9.80	3.72	0.03	2.55	11.26	0.21	2.57	0.61	0.11	301.3	12.74
MC	54.26	12.41	4.60	0.10	2.23	9.77	1.00	3.26	0.58	0.14	230.5	11.90
Sludge Ash	53.32	4.31	2.42	0.04	1.00	26.45	1.43	0.72	0.33	8.59	--	--
Bagasse Ash	44.33	0.29	0.97	0.13	4.60	6.30	0.24	2.89	0.05	21.29	0.10	--
Diatomaceous Earth Ash	67.34	4.95	0.66	--	0.11	0.43	1.20	1.31	0.27	0.35	0.09	0.04

**Table 2:** Total content C, H, H and S of raw materials.

Wastes	% C	% H	% N	% S	LHV (kcal/kg)	HHV (kcal/kg)
MC	2.140 ± 0.012	0.340 ± 0.001	0.025 ± 0.002	---	---	--
Bagasse	48.110 ± 0.070	7.570 ± 0.019	4.800 ± 0.025	---	4363.91 ± 47.13	4762.47 ± 47.13
Sludge	13.613 ± 0.192	1.973 ± 0.022	1.807 ± 0.046	---	1000.40 ± 1.87	1242.59 ± 1.87
Diatomaceous Earth	6.460 ± 0.036	0.876 ± 0.015	1.117 ± 0.001	---	583.19 ± 22.34	537.07 ± 22.34

**Table 3:** Loss on ignition in sintered materials.

T ^a^, °C	900 °C			950 °C			1000 °C		
Weight %	Loss on Ignition, %			Loss on Ignition, %			Loss on Ignition, %		
	Bagasse	Sludge	Diatomaceous	Bagasse	Sludge	Diatomaceous	Bagasse	Sludge	Diatomaceous
0	9.80	9.80	9.80	9.74	9.74	9.74	9.82	9.82	9.82
2	12.00	9.64	10.32	12.06	10.70	10.37	12.14	10.69	10.41
4	13.63	11.34	10.86	13.64	11.08	10.98	13.81	11.43	10.92
6	14.84	10.69	10.95	14.86	10.95	10.98	15.11	11.13	10.91
8	16.20	12.12	10.31	16.24	12.00	10.55	16.42	12.29	10.57
10	18.14	10.67	10.56	18.20	13.45	10.66	18.35	13.47	10.73
12.5	20.53	10.59	10.49	20.51	13.88	10.53	20.91	14.29	10.61
15	22.42	14.18	10.73	22.55	14.26	10.57	22.77	14.49	10.74

Note: T ^a^, sintering temperature in °C.

**Table 4:** Open porosity, bulk density and water absorption of sintered materials.

Clay	900 °C			950 °C			1000 °C		
	Open porosity, %	Water abs, %	Bulk density, Kg/m^3^	Open porosity, %	Water abs, %	Bulk density, Kg/m^3^	Open porosity, %	Water abs, %	Bulk density, Kg/m^3^
	35.06 ± 0.12	20.58 ± 0.14	1703.5 ± 08.7	34.57 ± 0.27	20.24 ± 0.25	1708.1 ± 10.3	35.5435 ± 0.6836	20.9253 ± 0.5088	1698.8 ± 09.0
**Brewery wastewater sludge**									
% w/w	Open porosity, %	Water abs, %	Bulk density, Kg/m^3^	Open porosity, %	Water abs, %	Bulk density, Kg/m^3^	Open porosity, %	Water abs, %	Bulk density, Kg/m^3^
2.0	31.65 ± 1.68	18.02 ± 0.96	1757.2 ± 26.4	33.84 ± 1.22	20.11 ± 0.73	1690.0 ± 21.7	36.03 ± 0.75	22.20 ± 0.49	1622.9 ± 10.7
4.0	35.86 ± 0.47	22.46 ± 0.36	1596.8 ± 07.6	36.52 ± 0.28	22.83 ± 0.28	1599.3 ± 09.0	36.88 ± 0.43	23.32 ± 0.39	1582.1 ± 09.6
6.0	35.36 ± 0.95	20.48 ± 0.70	1726.8 ± 18.9	32.59 ± 0.58	18.90 ± 0.41	1724.0 ± 12.7	29.83 ± 0.20	17.33 ± 0.13	1721.2 ± 06.4
8.0	38.69 ± 0.27	25.61 ± 0.27	1510.1 ± 06.5	40.19 ± 0.50	26.74 ± 0.43	1502.9 ± 07.0	47.08 ± 0.41	34.70 ± 0.32	1357.0 ± 05.3
10.0	41.26 ± 0.27	27.92 ± 0.26	1478.4 ± 07.1	42.69 ± 0.24	29.10 ± 0.33	1467.1 ± 11.0	39.84 ± 0.30	26.74 ± 0.20	1489.6 ± 03.2
12.5	38.23 ± 0.29	23.45 ± 0.26	1630.3 ± 06.8	41.47 ± 0.32	26.90 ± 0.24	1541.5 ± 05.2	46.97 ± 0.39	32.30 ± 0.35	1453.8 ± 05.2
15.0	42.57 ± 0.27	29.17 ± 0.27	1459.2 ± 07.4	43.79 ± 0.47	30.56 ± 0.48	1434.4 ± 09.1	45.01 ± 0.72	31.94 ± 0.68	1409.5 ± 10.7
**Bagasse**									
% w/w	Open porosity, %	Water abs, %	Bulk density, Kg/m^3^	Open porosity, %	Water abs, %	Bulk density, Kg/m^3^	Open porosity, %	Water abs, %	Bulk density, Kg/m^3^
2.0	36.77 ± 1.15	23.33 ± 0.81	1576.3 ± 13.9	37.20 ± 0.23	23.75 ± 0.30	1566.7 ± 12.9	37.63 ± 0.99	24.17 ± 0.79	1557.0 ± 12.7
4.0	42.41 ± 0.34	28.53 ± 0.36	1486.6 ± 08.5	42.86 ± 0.44	29.29 ± 0.39	1463.7 ± 09.6	43.30 ± 0.61	30.06 ± 0.49	1440.7 ± 04.3
6.0	45.36 ± 0.25	32.10 ± 0.32	1413.4 ± 06.8	46.33 ± 0.16	33,13 ± 0.31	1398.6 ± 08.5	49.98 ± 0.61	34.24 ± 0.73	1372.3 ± 12.4
8.0	48.29 ± 0.29	36.52 ± 0.35	1322.4 ± 06.7	49.29 ± 0.55	37.94 ± 0.46	1300.1 ± 14.3	50.29 ± 0.21	39.36 ± 0.28	1277.8 ± 08.0
10.0	51.33 ± 0.29	41.03 ± 0.41	1251.0 ± 06.2	52.24 ± 0.41	42.10 ± 0.62	1241.0 ± 08.6	51,61 ± 0,25	41.70 ± 0.31	1237.8 ± 07.5
12.5	54.73 ± 0.32	47.46 ± 0.39	1153.3 ± 11.2	55.01 ± 0.22	47.43 ± 0.36	1159.8 ± 05.7	54.46 ± 0.38	47.49 ± 0.43	1146.8 ± 08.9
15.0	56.25 ± 0.65	50.54 ± 1.14	1113.2 ± 13.3	56.54 ± 0.38	50.88 ± 0.30	1111.5 ± 10.2	56.84 ± 0.52	51.22 ± 0.61	1109.8 ± 04.2
**Diatomaceous earth**									
% w/w	Open porosity, %	Water abs, %	Bulk density, Kg/m^3^	Open porosity, %	Water abs, %	Bulk density, Kg/m^3^	Open porosity, %	Water abs, %	Bulk density, Kg/m^3^
2.0	36.77 ± 0.61	22.99 ± 0.36	1598.7 ± 05.8	36.68 ± 0.62	22.97 ± 0.40	1597.1 ± 07.1	36.59 ± 0.64	22.93 ± 0.44	1595.4 ± 08.3
4.0	40.32 ± 0.58	26.23 ± 0.45	1537.3 ± 09.0	40.14 ± 0.59	26.30 ± 0.48	1526.7 ± 09.2	39.97 ± 0.59	26.37 ± 0.50	1516.1 ± 09.3
6.0	41.29 ± 0.56	27.87 ± 0.59	1481.7 ± 11.5	42.56 ± 0.53	29.27 ± 0.62	1454.2 ± 12.5	44.11 ± 0.47	30.94 ± 0.52	1425.6 ± 09.0
8.0	43.77 ± 0.46	30.735 ± 0.48	1424.1 ± 08.0	44.73 ± 0.40	31.80 ± 0.46	1407.3 ± 08.4	45.70 ± 0.34	32.87 ± 0.43	1390.4 ± 08.7
10.0	44.59 ± 0.38	31.72 ± 0.42	1405.7 ± 07.1	45.61 ± 0.42	32.83 ± 0.43	1390.0 ± 07.4	46.63 ± 0.46	33.93 ± 0.44	1374.3 ± 07.6
12.5	47.37 ± 0.55	35.41 ± 0.73	1338.1 ± 12.5	47.96 ± 0.29	36.26 ± 0.45	1323.0 ± 08.6	48.10 ± 0.11	36.22 ± 0.23	1328.0 ± 06.3
15.0	50.13 ± 0.55	39.70 ± 0.86	1263.0 ± 14.5	50.37 ± 0.41	40.01 ± 0.63	1259.0 ± 11.4	50.61 ± 0.26	40.33 ± 0.41	1255.0 ± 08.2

**Table 5:** CFP of lightweight aggregates raw material extraction and sintering process in comparison with clay and 15% mass of wastes sintered at 1000 °C in terms of kg CO_2_ eq. contribution.

Impact Category	Unit	Sludge	Bagasse	Diatomaceous	Clay
IPCC GWP 20y	kg CO_2_ eq.	3.992	3.280	4.007	5.611
